# The efficacy of teriflunomide in patients who received prior disease-modifying treatments: Subgroup analyses of the teriflunomide phase 3 TEMSO and TOWER studies

**DOI:** 10.1177/1352458517695468

**Published:** 2017-03-17

**Authors:** Mark S Freedman, Jerry S Wolinsky, Giancarlo Comi, Ludwig Kappos, Tomas P Olsson, Aaron E Miller, Karthinathan Thangavelu, Myriam Benamor, Philippe Truffinet, Paul W O’Connor, for the TEMSO and TOWER Study Groups

**Affiliations:** University of Ottawa and The Ottawa Hospital Research Institute, The Ottawa Hospital, Ottawa, ON, Canada; Multiple Sclerosis Research Group and MRI-Analysis Center, McGovern Medical School, UTHealth, Houston, TX, USA; Department of Neurology, University Vita-Salute San Raffaele, Milan, Italy; Neurology and Department of Biomedicine, University Hospital Basel, Basel, Switzerland; Department of Clinical Neuroscience, Karolinska Institute, Stockholm, Sweden; The Corinne Goldsmith Dickinson Center for Multiple Sclerosis, Icahn School of Medicine at Mount Sinai, New York, NY, USA; Sanofi Genzyme, Cambridge, MA, USA; Sanofi Genzyme, Chilly-Mazarin, France; Sanofi Genzyme, Chilly-Mazarin, France; University of Toronto, Toronto, ON, Canada

**Keywords:** Teriflunomide, disease-modifying treatment, TEMSO, TOWER, RRMS, ARR and disability worsening

## Abstract

Teriflunomide is a once-daily oral immunomodulator approved for relapsing-remitting multiple sclerosis (MS). The objective of this post hoc analysis of the phase 3, pooled TEMSO (NCT00134563) and TOWER (NCT00751881) dataset is to evaluate the effect of teriflunomide treatment on annualised relapse rate and disability worsening across patient subgroups defined according to prior disease-modifying therapy exposure. This analysis provides further supportive evidence for a consistent effect of teriflunomide across a broad range of patients with relapsing MS, including patients who have used and discontinued other disease-modifying therapies.

## Introduction

In patients with multiple sclerosis (MS), treatment switches are often considered to improve adherence, address a lack of efficacy or mitigate tolerability and/or safety concerns.^1,2^ The decision to change therapy is complex, requiring assessment of therapeutic benefit–risk and the potential for further disease worsening.^3^ Patients who have previously received treatment and require multiple changes in therapy may have more active disease and be at increased risk of relapse or disease worsening compared with treatment-naïve patients.^4^

Teriflunomide is a once-daily oral immunomodulator approved for relapsing-remitting multiple sclerosis (RRMS) that has been evaluated in two pivotal phase 3 studies: TEMSO (NCT00134563) and TOWER (NCT00751881).^5,6^ In the individual studies and pooled dataset, teriflunomide 14 mg significantly reduced annualised relapse rate (ARR) and risk of disability worsening confirmed for 12 weeks compared with placebo.^5,6^ In addition, safety and tolerability profiles for teriflunomide were similar within the individual studies and pooled analyses.^5–7^

TEMSO and TOWER included patients who received one or more other disease-modifying therapies (DMTs) in the 2 years prior to study entry (but had not used them within the 3–6 months before randomisation).^5,6,8^ We evaluated ARR and disability worsening in patients exposed to prior treatment versus patients who had not received a prior DMT in the previous 2 years, described herein as ‘treatment-naïve’.

## Methods

TEMSO and TOWER were multicentre, multinational, randomised (1:1:1 to once-daily oral placebo or teriflunomide 7 or 14 mg), double-blind, parallel-group, placebo-controlled phase 3 studies.^5,6^ Duration of treatment was fixed in TEMSO (108 weeks),^5^ and varied in TOWER (48–173 weeks), where the study ended 48 weeks after last patient randomised.^6^ Both studies enrolled adults (18–55 years) with relapsing forms of MS and Expanded Disability Status Scale (EDSS) scores ≤5.5. Patients were required to have ≥1 relapse (12 months) or ≥2 relapses (24 months) before study entry.^5,6^ Both TEMSO and TOWER included patients who had received one or more DMT in the 2 years prior to study entry but had not used them within 3–6 months before randomisation.^5,6,8^

Post hoc analyses were performed on the pooled, modified intent-to-treat population from both studies (all patients randomised who received ≥1 dose of study medication), in subgroups defined according to DMT exposure in the previous 2 years: ≥2 prior DMTs, 1 prior DMT or no prior DMT. Reasons for discontinuation or switch of prior therapy were not recorded, but may have been due to perceived sub-optimal treatment response, poor adherence, or safety and tolerability issues. Outcomes included ARR and disability worsening confirmed for 12 weeks (defined as an increase from baseline of ≥1.0 EDSS point (or ≥0.5 points for a baseline EDSS score >5.5) for at least 12 weeks). Magnetic resonance imaging (MRI) was not performed in TOWER and thus is not included as an outcome in this analysis.

### Statistical analysis

ARR was derived from an analysis of the number of relapses, using a Poisson regression model with the log of time during treatment as an offset variable. For disability worsening, a log-rank test was used to compare teriflunomide with placebo, and a hazard ratio was estimated using a Cox regression model. For all endpoints, analysis models were adjusted for treatment, EDSS strata at baseline (≤3.5 or >3.5), region, study and prior-treatment subgroup as covariates. Consistency of treatment effect across prior-treatment subgroups was evaluated using a treatment-by-subgroup interaction term. Inferential analyses were performed at the two-sided 5% level of significance.

## Results

In total, 2251 patients were included in the pooled analysis. Baseline disease characteristics of the pooled TEMSO/TOWER population according to prior DMT were generally well balanced across the three groups; however, disease duration was shorter in the treatment-naïve population (Table 1). Most patients were treatment-naïve, with ~30% having used one or more prior DMT. Across all treatment groups, the most frequently used prior DMT was interferon beta (IFNβ) (Table 1).

**Table 1.** Baseline disease characteristics by number of prior treatments and prior treatment by treatment group (mITT population).

	Pooled dataset (*N* = 2251)		
	≥2 prior DMT	1 prior DMT	No prior DMT
Patients, *n* (%)	109 (4.8)	574 (25.5)	1568 (69.7)
Age, mean (SD), years	38.7 (8.2)	37.6 (8.8)	38.0 (9.2)
Sex, female, *n* (%)	96 (88.1)	427 (74.4)	1089 (69.5)
Weight, mean (SD), kg	72.7 (18.6)	71.8 (17.6)	70.1 (15.7)
Years since first diagnosis of MS, mean (SD)	7.34 (4.89)	7.04 (5.52)	4.44 (5.46)
Years since first symptoms of MS, mean (SD)	10.26 (6.19)	9.67 (6.31)	7.70 (6.95)
Months since most recent relapse onset, mean (SD)	6.39 (4.09)	6.10 (3.59)	5.64 (3.41)
Relapses in past year, median (min:max)	1.0 (0:4)	1.0 (0:6)	1.0 (0:7)
Relapses in past 2 years, median (min:max)	2.0 (1:9)	2.0 (1:12)	2.0 (1:8)
Relapsing-remitting MS, %	97.2	95.6	94.0
Baseline EDSS score, median (min:max)	2.5 (0.0:5.5)	2.5 (0.0:6.0)	2.5 (0.0:6.5)
	Pooled dataset (*N* =2251)^a^		
	Placebo (*n* =751)	Teriflunomide 7 mg (*n* =772)	Teriflunomide 14 mg (*n* =728)
No prior treatment within 2 years prior to study	523 (69.6)	547 (70.9)	498 (68.4)
Prior DMT (as reported in CRF)			
IFNβ total	167 (22.2)	178 (23.1)	179 (24.6)
IFNβ-1a	117 (15.6)	137 (17.7)	126 (17.3)
IFNβ-1a SC	73 (9.7)	82 (10.6)	73 (10.0)
IFNβ-1a IM	49 (6.5)	65 (8.4)	57 (7.8)
IFNβ-1a unspecified	4 (0.5)	3 (0.4)	5 (0.7)
IFNβ-1b	56 (7.5)	49 (6.3)	62 (8.5)
IFNβ	5 (0.7)	2 (0.3)	4 (0.5)
GA	88 (11.7)	70 (9.1)	80 (11.0)
Fingolimod	1 (0.1)	2 (0.3)	4 (0.5)
Natalizumab	1 (0.1)	0	0

CRF: case record form; DMT: disease-modifying therapy; EDSS: Expanded Disability Status Scale; GA: glatiramer acetate; IFNβ: interferon beta; IM: intramuscular; mITT: modified intent-to-treat; MS: multiple sclerosis; SC: subcutaneous; SD: standard deviation.

a Data provided as *n* (%); patients within each group may have used more than one listed DMT.

### ARR

**Figure 1.**
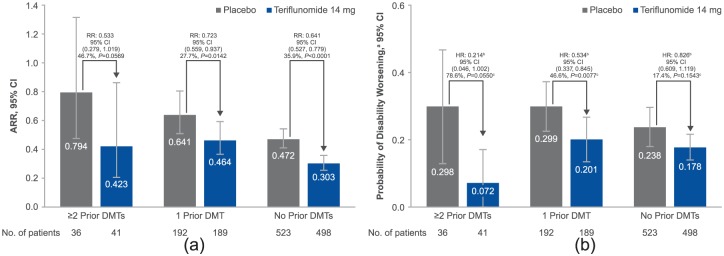
(a) ARR by prior treatment. Overall *p* value for treatment-by-subgroup interaction for ARR: 14 mg, *p* = 0.4344. Percentages represent relative risk reductions (95% CI). ARR (95% CI) for teriflunomide 7 mg: ≥2 prior DMTs, 0.463 (0.231, 0.930), RR (95% CI) 0.584 (0.308, 1.104), difference versus placebo 41 .6%, *p* =0.0977; 1 prior DMT, 0.536 (0.423, 0.680), RR (95% CI) 0.836 (0.643, 1.087), difference versus placebo 16.4%, *p* =0.1804; no prior DMT, 0.329 (0.285, 0.380), RR (95% CI) 0.698 (0.583, 0.835), difference versus placebo 30.2%, *p* =0.0001. Overall *p* value for treatment-by-subgroup interaction for ARR, *p* =0.3947. In the placebo arms, ARR was significantly higher for patients with ≥2 DMTs (*p* =0.0183) or 1 prior DMT (*p* =0.0008) compared with treatment-naïve patients. (b) Disability worsening by prior treatment. ^a^Derived from Kaplan–Meier estimates at week 132. ^b^Derived using a Cox proportional hazard model with treatment, EDSS strata at baseline, region and study as covariates. ^c^Derived from log-rank test, with EDSS strata at baseline, region, study and subgroup as covariates. Overall *p* value for treatment-by-subgroup interaction for disability worsening: 14 mg, *p* =0.0697. Percentages represent relative risk reductions (95% CI) on the hazard ratios. Probability of disability worsening^a^ (95% CI) for teriflunomide 7 mg: ≥2 prior DMTs, 0.218 (0.061, 0.376), HR (95% CI) 0.666 (0.223, 1.992), difference versus placebo 33.4%, *p* =0.5707^c^; 1 prior DMT, 0.345 (0.259, 0.431), HR (95% CI) 0.950 (0.634, 1.422), difference versus placebo 5.0%, *p* =0.8505^c^; no prior DMT, 0.176 (0.139, 0.212), HR (95% CI) 0.792 (0.587, 1.068), difference versus placebo 20.8%, *p* =0.0948^c^. Overall *p* value for treatment-by-subgroup interaction for disability worsening: 7 mg, *p* =0.6921. In the placebo arms, there was a significantly greater risk of disability progression in patients with 1 prior DMT (*p* =0.0250) compared with treatment-naïve patients. The risk was also greater in patients with ≥2 prior DMTs, although significance was not reached (*p* = 0.4486). ARR: annualised relapse rate; CI: confidence interval; DMT: disease-modifying therapy; EDSS: Expanded Disability Status Scale; HR: hazard ratio; RR: relative risk.

Compared with treatment-naïve patients, ARRs were higher for patients in the placebo group exposed to either ≥2 prior DMTs or 1 prior DMT (Figure 1(a)). Across all subgroups, a greater reduction in ARR was reported following teriflunomide treatment versus placebo. Consistency of treatment effect of teriflunomide 14 mg is supported by the non-significant treatment-by-subgroup interaction (*p* = 0.4344). Reductions in ARR with teriflunomide were numerically greater in patients who had received ≥2 prior DMTs versus those who had used 1 prior DMT.

### Disability worsening

Placebo-treated patients had a numerically greater risk of disability worsening in both prior-treatment subgroups compared with treatment-naïve patients (Figure 1(b)). Consistency of treatment effect of teriflunomide 14 mg was established with a non-significant treatment-by-subgroup interaction, although this trended towards significance (*p*=0.0697). The treatment effect of teriflunomide 14 mg on reducing risk of disability worsening was numerically greater in patients who had received ≥2 prior DMTs or 1 prior DMT versus treatment-naïve patients.

### MRI outcomes (TEMSO only)

As noted for the clinical outcomes, placebo-treated patients in prior DMT groups tended to have higher MRI activity with more enhancing lesions (Supple-mental Figure 1(a)), although teriflunomide positively impacted MRI activity across all subgroups regardless of prior exposure (Supplemental Figure 1(b)).

## Discussion

In this analysis of the TEMSO/TOWER pooled data set, teriflunomide 14 mg was associated with reductions in ARR and risk of disability worsening across all subgroups defined by prior DMT exposure compared with placebo. Due to small group sizes limiting statistical power, treatment effect did not reach significance in all instances; however, the direction of change on both outcomes was the same regardless of prior DMT exposure. In the placebo arms, patients with prior DMT exposure were at higher risk of relapses and disability worsening than treatment-naïve patients. Nevertheless, for both ARR and disability worsening, reductions with teriflunomide treatment were numerically greater in patients with prior DMT exposure. These results also help to address prior perceptions that patients who require switching from their current DMT are at higher risk of relapse or disability worsening in the imminent future.^4^ This risk was consistently greater in placebo-treated patients who had one or more prior DMT versus treatment-naïve patients.

Although there are limitations to this analysis (e.g. small group sizes and lack of information on reasons for switching from prior DMTs), these pooled subgroup analyses support the efficacy of teriflunomide across a broad range of patients with RRMS, including those who have discontinued previous DMTs – a subgroup of patients that can be challenging to treat.

## Supplementary Material

Supplementary material
