# Supplementary material for: The efficacy of teriflunomide in patients who received prior disease-modifying treatments: Subgroup analyses of the teriflunomide phase 3 TEMSO and TOWER studies
Source: Mult Scler. 2017 Mar 17;24(4):535–9. doi: 10.1177/1352458517695468 (PMC5891690; doi:10.1177/1352458517695468)
Supplement: Supplementary material [file MSJ695468_supplementary_material.pdf]

Supplementary material

Magnetic resonance imaging statistical analysis

MRI lesion count data was also analyzed using a Poisson regression model with treatment, Expanded Disability Status Scale strata at baseline, region and baseline number of Gd+ T1 lesions as covariates, and log-transformed number of scans as an offset variable.

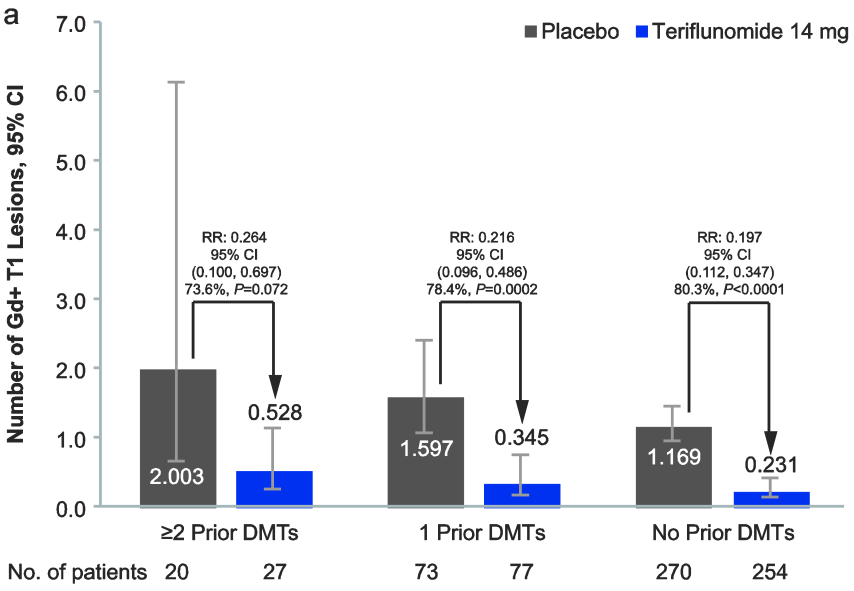

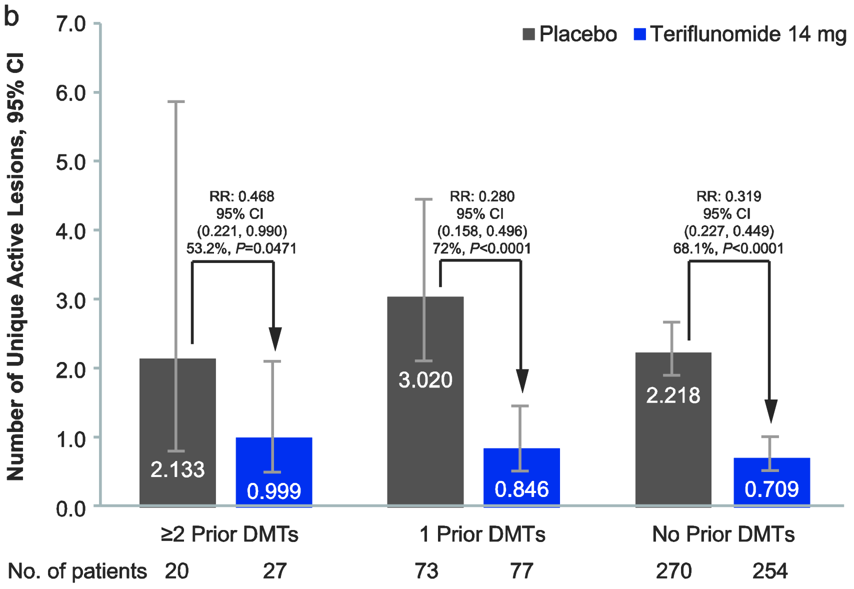

Supplemental Figure 1a. Gd-enhancing T1 lesions by prior treatment

Overall *P* value for treatment-by-subgroup interaction for Gd-enhancing (Gd+) T1 lesions: 14 mg, *P*=0.4627. Percentages represent relative risk reductions (95% CI).

Number of Gd+ T1 lesions (95% CI) for teriflunomide 7 mg: ≥2 prior DMTs, 1.316 (0.544, 3.180), RR (95% CI) 0.657 (0.242, 1.782), difference versus placebo 34.3%, *P*=0.4089; 1 prior DMT, 0.672 (0.370, 1.222), RR (95% CI) 0.421 (0.221, 0.802), difference versus placebo 57.9%, *P*=0.0085; no prior DMT, 0.561 (0.404, 0.779), RR (95% CI) 0.480 (0.338, 0.681), difference versus placebo 52.0%, *P*<0.0001. Overall *P* value for treatment-by-subgroup interaction for Gd+ T1 lesions, *P*=0.3983.

In the placebo arms, there was a significantly higher number of Gd+ T1 lesions in patients with 1 prior DMT (*P*=0.0211) compared with treatment-naïve patients. The number of Gd+ T1 lesions was also higher in patients with ≥2 prior DMTs, although significance was not reached (*P*=0.8907).

CI, confidence interval; DMT, disease-modifying therapy; Gd, gadolinium; RR, relative risk.

Supplemental Figure 1b. Unique active lesions by prior treatment

Overall *P* value for treatment-by-subgroup interaction for unique active lesions: 14 mg, *P*=0.4254. Percentages represent relative risk reductions (95% CI).

Number of unique active lesions (95% CI) for teriflunomide 7 mg: ≥2 prior DMTs, 2.076 (0.946, 4.557), RR (95%CI) 0.973 (0.412, 2.294), difference vs placebo 2.7%, *P*=0.9508; 1 prior DMT, 1.381 (0.879, 2.169), RR (95% CI) 0.457 (0.291, 0.718), difference vs placebo 54.3%, *P*=0.0007; no prior DMT, 1.243 (1.000, 1.544), RR (95% CI) 0.560 (0.440, 0.714), difference versus placebo 44%, *P*<0.0001. Overall *P* value for treatment-by-subgroup interaction for unique active lesions, *P*=0.1648.

In the placebo arms, the number of unique active lesions was significantly higher for patients with 1 prior DMT (*P*=0.0120) compared with treatment-naïve patients. The number of unique active lesions was also higher for patients with ≥2 prior DMTs, although significance was not reached (*P*=0.4983).

CI, confidence intervals; DMT, disease-modifying therapy; RR, relative risk.
